# Analysis of the time-to-onset of osteonecrosis of jaw with bisphosphonate treatment using the data from a spontaneous reporting system of adverse drug events

**DOI:** 10.1186/s40780-015-0035-2

**Published:** 2015-12-22

**Authors:** Mitsuhiro Nakamura, Ryogo Umetsu, Junko Abe, Toshinobu Matsui, Natsumi Ueda, Yamato Kato, Sayaka Sasaoka, Kohei Tahara, Hirofumi Takeuchi, Yasutomi Kinosada

**Affiliations:** Laboratory of Drug Informatics, Gifu Pharmaceutical University, 1-25-4 Daigaku-nishi, Gifu, 501-1196 Japan; Medical Database Co., LTD, 3-11-10 Higashi, Shibuya-ku, Tokyo 150-0011 Japan; Laboratory of Pharmaceutical Engineering, Gifu Pharmaceutical University, 1-25-4 Daigaku-nishi, Gifu, 501-1196 Japan; United Graduate School of Drug Discovery and Medical Information Sciences, Gifu University, 1-1 Yanagido, Gifu, 501-1194 Japan; Present address: Clinical Research, Innovation and Education Center, Tohoku University Hospital, 1-1 Seiryo-machi, Aoba Ward, Sendai, Miyagi 980-8574 Japan

**Keywords:** Bisphosphonate, Osteonecrosis of jaw, Japanese adverse drug event report database

## Abstract

**Background:**

Bisphosphonates (BPs) are potent antiresorptive agents used to treat osteoporosis and the complications associated with malignant bone metastasis. The aim of this study was to evaluate the incidence of bisphosphonate-related osteonecrosis of the jaw (BRONJ) using the Japanese Adverse Drug Event Report (JADER) database. In particular, we focused on the time-to-onset profile of BRONJ.

**Findings:**

We analyzed reports of BRONJ in the JADER database and calculated the reporting odds ratio (ROR) of BPs potentially associated with BRONJ. We applied the weibull shape parameter to time-to-event data in JADER. The drugs selected for this investigation were seven BPs approved in Japan (alendronate [intraveneous, I.V.], pamidronate, and zoledronate as I.V. BPs; and alendronate (oral), etidronate, minodronate, and risedronate as oral BPs). We analyzed reports of BRONJ events associated with BPs in the JADER database from April 2004 to November 2014. The median value of BRONJ cases caused by alendronate (I.V.), pamidronate, zoledronate, alendronate (oral), etidronate, minodronate, and risedronate were 1342, 812, 486, 863, 1461, 432, and 730 days, respectively. The lower 95 % confidence interval of the Weibull-shape parameter β for I.V. BPs (pamidronate and zoledronate) exceeded 1. The risk of BRONJ with I.V. BPs increased over time.

**Conclusion:**

Thus, the incidence of BRONJ with BP treatment should be closely monitored for a 3-year period. Further studies are required to draw conclusions, and we believe that this information about BRONJ induced by BPs will prove beneficial to patients and pharmacists.

## Findings

### Background

Bisphosphonates (BPs) are widely used for the treatment of osteoporosis and complications associated with malignant bone metastasis [1]. Bisphosphonate-related osteonecrosis of the jaw (BRONJ) has been recognized as an uncommon but severe adverse event that affects the quality of life of patients. The risk of BRONJ depends on the type of BPs and the duration of exposure, and the majority of BRONJ patients experience the complication following simple dentoalveolar surgery [2, 3]. According to the position paper on BRONJ from American Association of Oral and Maxillofacial Surgeons (AAOMS), patients receiving oral BPs are at a risk of BRONJ, but to a much lesser degree than those treated with I.V. BPs. The risk of oral BPs may be associated with longer treatment durations, such as over 3–4 years [2, 3].

Since BRONJ is a rare adverse event [4], epidemiologic research on this condition is difficult. The regulatory authority in Japan, the Pharmaceuticals and Medical Devices Agency (PMDA), has released the spontaneous reporting system (SRS) of the Japanese Adverse Drug Event Report (JADER) database. The JADER database is the largest and best-known database, and it reflects the realities of clinical practice. Therefore, JADER has been recognized as one of the primary tools for pharmacovigilance assessments [5]. Analysis of time-to-onset data has been proposed as a method to detect signals for adverse drug reactions (ADRs) in SRS [6–8]. As far as we know, analyses of the time-to-onset for BRONJ using the JADER database are rare. The aim of this study was to assess the time-to-onset data of BRONJ by BP treatment.

### Methods

**Fig. 1:**
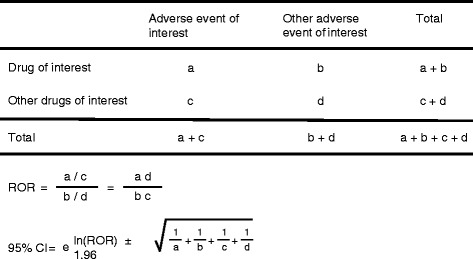
Two by two table used for the calculation of reporting odds ratios

Adverse events recorded from April 2004 to November 2014 in the JADER database were downloaded from the PMDA website (http://www.pmda.go.jp). The database consists of 4 data tables: patient demographic information (demo), drug information (drug), adverse events (reac), and primary disease (hist). The adverse events in “reac” are coded according to the terminology preferred by the Medical Dictionary for Regulatory Activities (MedDRA) [9]. “Drug” file (drug information) includes role codes assigned to each drug: suspected drug (higiyaku in Japanese), interacting drug (sougosayou in Japanese), and concomitant drug (heiyouyaku in Japanese). Suspected drugs were extracted and analyzed in this study. The drugs selected for this investigation were seven BPs approved in Japan (alendronate [intraveneous, I.V.], pamidronate, and zoledronate as I.V. BPs; alendronate (oral), etidronate, minodronate, and risedronate as oral BPs). BPs were grouped by dosages and each BP was stratified by dosages: pamidoronate (30–45 mg), pamidoronate (90 mg), zoledronate (4 mg), alendronate (5 mg), alendronate (35 mg), risedronate (2.5 mg), and risedronate (17.5 mg). We used the preferred term “osteonecrosis of jaw” to match cases of BRONJ according to MedDRA. To detect the incidence of BRONJ, we calculated the reporting odds ratio (ROR), which is an established parameter for pharmacovigilance research, using a disproportionality analysis [10]. “Cases” were defined as patients who reported BRONJ, while “non-cases” consisted of patients associated with all other reports. The ROR is the ratio of the odds of reporting adverse events versus all other events associated with BPs compared to the reporting odds for all other drugs present in the database. To compare the “cases” and “non-cases,” we calculated the RORs as (a:c)/(b:d) (Fig. 1). RORs were expressed as point estimates with a 95 % confidence interval (CI). For signal detection, general qualitative judgments were used. The detection of a signal was dependent on the signal indices exceeding a predefined threshold. ROR values <1 indicated no exposure-event association, and estimates >1 indicated exposure-event safety signals. Safety signals are considered significant when the ROR estimates and the lower limits of the corresponding 95 % CI are >1 [10].

The median of the data, quartiles, and the Weibull shape parameter (WSP) were utilized in evaluations of the time-to-onset data for BRONJ. Time-to-onset from the JADER database was calculated from the beginning of the time of a subject’s first prescription to the occurrence of the adverse events. Different BPs were not administered at the same time. For duplicate entries of the same BPs in the same case, we adopted the most recent BP entry in order to identify the beginning of the time of a subject’s first prescription. The WSP test is used for statistical analysis of time-to-onset data and can describe the non-constant rate of incidence of ADRs (i.e., the risk of increase or decrease over time) [7, 8]. The shape parameter β of the Weibull distribution indicated that the hazard without a reference population; when β is equal to 1, the hazard is estimated to be constant over time. If β is greater than 1 and the 95 % CI of β excluded the value 1, the hazard was considered to increase over time.

All data analyses were performed by JMP 11.0 (SAS Institute Inc., Cary, NC, USA).

### Results

The JADER database contains 338,224 reports from April 2004 to November 2014. The database contains 4,128,716 combinations of drugs and ADRs. After extracting the combinations completed within the beginning date of the treatment and the occurrence date of adverse events, a total of 1,777,925 combinations were analyzed. The number of reports with “osteonecrosis of jaw” was 3,027. The RORs (95 % CI) of alendronate (I.V.), pamidronate, zoledronate, alendronate (oral), etidronate, minodronate, and risedronate were 43.7 (22.1–86.4), 315.0 (256.3–387.0), 346.2 (308.2–388.8), 92.3 (81.4–104.5), 160.3 (83.5–307.7), 17.3 (12.7–23.5), and 48.4 (40.3–58.0), respectively (Table 1). The number of cases for zoledronate in males and females were 301 and 474, respectively (Table 1). The number of cases for alendronate (oral) in males and females were 22 and 379, respectively.

**Table 1:** Number of reports and reporting odds ratio of BRONJ stratified by gender

Dosage form	Drug name		Case	Non-case	Total	Reporting odds ratio	(95 % CI)
Intravenous	Alendronate (I.V.)		10	48	58	43.7	(22.1 − 86.4)
		Male	2	8	10	52.1	(11.1 − 245.7)
		Famale	8	40	48	41.9	(19.6 − 89.6)
	Pamidronate		223	171	394	315.0	(256.3 − 387.0)
		Male	30	35	65	181.9	(111.4 − 297.0)
		Famale	186	128	314	341.9	(271.4 − 430.8)
	Zoledronate		809	972	1,781	346.2	(308.2 − 388.8)
		Male	301	436	737	176.5	(150.9 − 206.4)
		Famale	474	514	988	271.4	(236.5 − 311.4)
Oral	Alendronate (oral)		414	1,252	1,666	92.3	(81.4 − 104.5)
		Male	22	146	168	31.8	(20.3 − 49.9)
		Famale	379	1,096	1,475	93.8	(82.4 − 106.8)
	Etidronate		16	21	37	160.3	(83.5 − 307.7)
		Male	3	4	7	156.5	(35.0 − 699.9)
		Famale	13	17	30	160.6	(77.9 − 331.1)
	Minodronate		45	558	603	17.3	(12.7 − 23.5)
		Male	4	49	53	17.0	(6.1 − 47.3)
		Famale	41	483	524	18.1	(13.1 − 25.0)
	Risedronate		152	721	873	48.4	(40.3 − 58.0)
		Male	18	81	99	46.8	(28.0 − 78.2)
		Famale	131	614	745	48.3	(39.7 − 58.7)

The RORs stratified by dosages are summarized in Table 2. The complete report with dosage amount was analyzed. The RORs for pamidoronate (30–45 mg), pamidoronate (90 mg), zoledronate (4 mg), alendronate (5 mg), alendronate (35 mg), risedronate (2.5 mg), and risedronate (17.5 mg) were 159.4 (107.5–236.2), 298.0 (204.8–433.6), 226.6 (198.8–258.3), 91.7 (75.6–111.3), 68.2 (57.8–80.4), 42.5 (32.9–54.8), and 34.9 (24.3–50.1), respectively.

**Table 2:** Number of reports and reporting odds ratio of BRONJ stratified by formulations and dosages

Dosage form	Drug name		Case	Non-case	Total	Reporting odds ratio	(95 % CI)
Intravenous	Pamidronate		223	171	394	315.0	(256.3 − 387.0)
		30 mg − 45 mg^a^	44	59	103	159.4	(107.5 − 236.2)
		90 mg^a^	66	48	114	298.0	(204.8 − 433.6)
	Zoledronate		809	972	1,781	346.2	(308.2 − 388.8)
		4 mg^a^	494	651	1,145	226.6	(198.8 − 258.3)
Oral	Arendronate (oral)		414	1,252	1,666	92.3	(81.4 − 104.5)
		5 mg^a^	155	388	543	91.7	(75.6 − 111.3)
		35 mg^a^	202	702	904	68.2	(57.8 − 80.4)
	Risedronate		152	721	873	48.4	(40.3 − 58.0)
		2.5 mg^a^	73	374	447	42.5	(32.9 − 54.8)
		17.5 mg^a^	35	213	248	34.9	(24.3 − 50.1)

^a^Reports completed with dosage amount were analyzed

The number of case reports for alendronate (I.V.), pamidronate, zoledronate, alendronate (oral), etidronate, minodronate, and risedronate was 6, 118, 408, 196, 9, 32, and 65, respectively (Table 3). The median values for BRONJ caused by alendronate (I.V.), pamidronate, zoledronate, alendronate (oral), etidronate, minodronate, and risedronate were 1342, 812, 486, 863, 1461, 432, and 730 days, respectively (Table 3). The Weibull distribution parameters for each BP are summarized in Table 3. The lower 95 % CI of β for both I.V. BPs (pamidronate and zoledronate) and alendronate (oral) exceeded 1, but those for other BPs did not exceed 1 (Table 3). The median values for BRONJ caused by pamidronate (30–45 mg), pamidoronate (90 mg), zoledronate (4 mg), alendronate (5 mg), alendronate (35 mg), risedronate (2.5 mg), and risedronate (17.5 mg) were 844, 783, 488, 1263, 680, 1081, and 112 days, respectively (Table 4). The lower 95 % CI of β for pamidronate (30–45 mg), pamidoronate (90 mg), zoledronate (4 mg), alendronate (5 mg), and risedronate (2.5 mg) exceeded 1, but those for alendronate (35 mg) and risedronate (17.5 mg) did not exceed 1 (Table 4).

**Table 3:** Time-to-onset analysis of bisphosphonates

Dosage form	Drug name	Case (number for analysis)	Median of time-to-onset (day)	Lower quartile of time-to-onset (day)	Upper quartile of time-to-onset (day)	Scale parameter: α		Shape parameter: β	
						α	(95 % CI)	β	(95 % CI)
Intravenous	Alendronate (I.V.)	10 (6)	1,342	691	1,701	1267.6	(737.0 − 2125.0)	2.15	(0.96 − 3.83)
	Pamidronate	223 (118)	812	567	1,227	934.7	(816.9 − 1066.3)	1.45	(1.26 − 1.64)
	Zoledronate	809 (408)	486	274	821	631.5	(586.8 − 678.9)	1.41	(1.30 − 1.52)
Oral	Alendronate (oral)	414 (196)	863	432	1,463	1095.4	(970.2 − 1233.8)	1.23	(1.09 − 1.37)
	Etidronate	16 (9)	1,461	389	2,068	1390.3	(766.8 − 2442.7)	1.49	(0.74 − 2.63)
	Minodronate	45 (32)	432	196	742	629.5	(408.3 − 954.8)	0.90	(0.69 − 1.13)
	Risedronate	152 (65)	730	215	1,494	951.7	(730.3 − 1228.7)	1.02	(0.82 − 1.24)

**Table 4:** Time-to-onset analysis of bisphosphonates stratified by formulations and dosages

Dosage form	Drug name	Case (number for analysis)		Median of time-to-onset (day)	Lower quartile of time-to-onset (day)	Upper quartile of time-to-onset (day)	Scale parameter: α		Shape parameter: β	
							α	(95 % CI)	β	(95 % CI)
Intravenous	Pamidronate (30 − 45 mg)	44	(35)	844	658	1,199	775.6	(641.8 − 928.9)	1.98	(1.49 − 2.53)
	Pamidronate (90 mg)	66	(48)	783	532	1,041	889.5	(706.1 − 1112.6)	1.35	(1.09 − 1.63)
	Zoredronate (4 mg)	494	(355)	488	290	811	634.1	(587.7 − 683.4)	1.46	(1.34 − 1.59)
Oral	Arendronate (5 mg)	155	(72)	1,263	757	1,951	1450.5	(1258.1 − 1664.5)	1.77	(1.46 − 2.12)
	Arendronate (35 mg)	202	(102)	680	309	1,106	804.9	(663.0 − 972.3)	1.08	(0.91 − 1.25)
	Risedronate (2.5 mg)	73	(39)	1,081	495	1,706	1288.6	(1008.4 − 1634.3)	1.41	(1.06 − 1.82)
	Risedronate (17.5 mg)	35	(14)	112	33	407	295.0	(129.5 − 636.9)	0.82	(0.96 − 1.23)

**Fig. 2:**
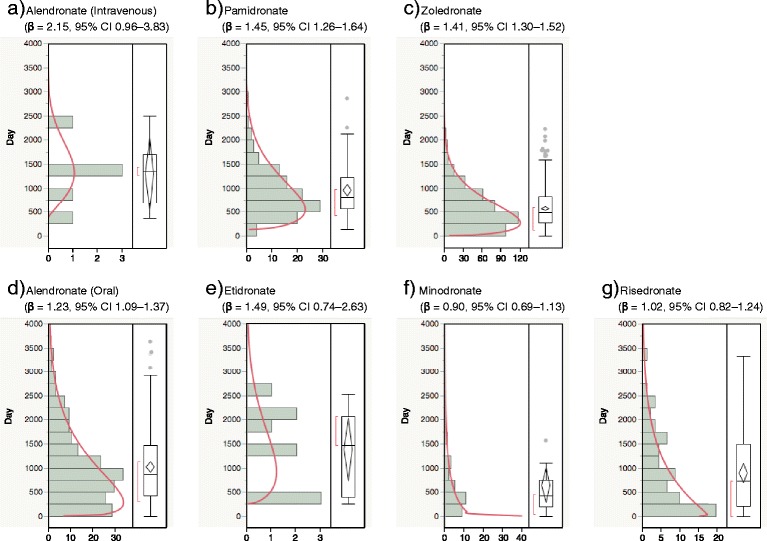
Histogram and Weibull shape parameter of BRONJ for (**a**) alendronate (Intravenous), (**b**) pamidronate, (**c**) zoledronate, (**d**) alendronate (oral), (**e**) etidronate, (**f**) minodronate and (**g**) risedronate

**Fig. 3:**
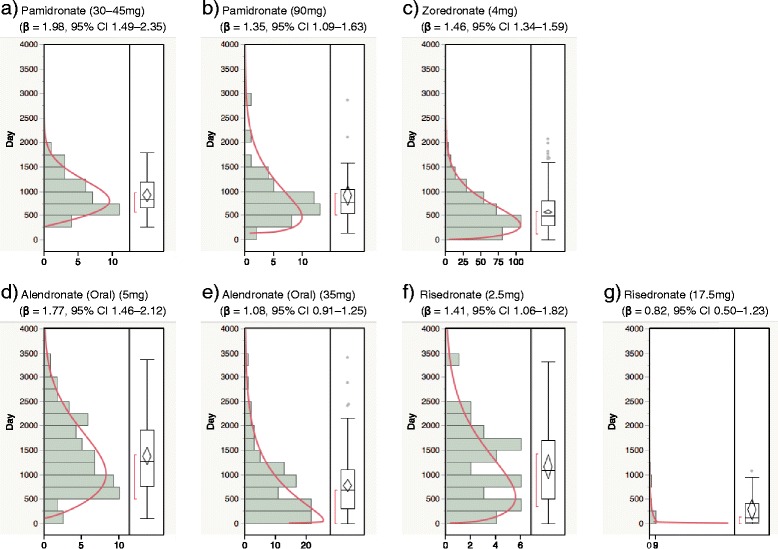
Histogram and Weibull shape parameter of BRONJ stratified by formulations and dosages for (**a**) pamidronate (30−45mg), (**b**) pamidronate (90mg), (**c**) zoredronate (4mg), (**d**) alendronate (oral) (5mg), (**e**) alendronate (oral) (35mg), (**f**) risedronate (2.5mg) and (**g**) risedronate (17.5mg)

Figures 2 and 3 show the histogram of the number of cases of BRONJ between 0 day to 4,000 days. The interval of the quartile points was very large in all BPs, but the lower quartile point was over 150 days for all BPs except risedronate (17.5 mg).

### Discussion

The risks of BRONJ related to antiresorptive therapy are well recognized by clinicians [11]. In patients receiving drugs via the I.V. route, the prevalence of BRONJ was 1–10 %, while the prevalence was 0.00007–0.04 % in those using the oral route [3]. The I.V. route of administration results in a greater drug exposure than the oral route [2, 3]. Zoledronate is more potent than pamidronate, while pamidronate is more potent than oral BPs [2, 3]. In *in vitro* analysis, zoledronate was the most potent inhibitor of farnesyl pyrophosphate synthase, followed by risedronate, alendronate, and pamidronate [12]. The inhibition of farnesyl pyrophosphate is associated with effectiveness in suppressing bone turnover *in vivo* [13]. Several reports have suggested that the pathogenesis of BRONJ is partially associated with osteonecrosis by suppression of bone turnover. In our study, the number of reported cases of BRONJ increased after administration of BPs. The obtained RORs for all of the BPs and their lower limits of 95 % CI were >1. The RORs for zoledronate and pamidronate were higher than those for the oral BPs, and the ROR for zoledronate was higher than that for alendronate (I.V.). The results obtained in this study were reasonable in the context of those reported in the literature from the JADER database.

The risk of developing BRONJ associated with oral BPs increases when the duration of therapy exceeds 3–4 years [2, 3]. The median duration of BP exposure for patients with BRONJ and BRONJ-like features was 4.4 years [14, 15]. Yoneda *et al.* demonstrated that the incidence of BRONJ begins to increase 1 year after intravenous BP and 2–3 years after oral BP administration [11]. In Japan, the mean duration of administration until onset of BRONJ was 23.6 months (2.0 years) for I.V. BPs and 33.3 months (2.8 years) for oral BPs [16]. The median time-to-onset of BRONJ for BPs, zoledronate, pamidronate, and alendronate (oral) was 486 (1.3 years), 812 (2.2 years), and 863 days (2.4 years), respectively. BRONJ with BPs treatment should be closely monitored within 3 years. The results of the shape parameter of the Weibull distribution indicated that the risk of BRONJ with the oral BPs, minodronate and risedronate, is almost constant. The number of case reports for analysis of etidronate, minodronate, and risedronate were less than 100 (Table 3). Owing to the low frequency of disease, studies with small samples need to be interpreted cautiously. However, the risk of BRONJ with I.V. BPs, pamidronate and zoledronate, increased over time. This is likely because of the strong exposure to I.V. BPs among the patients. More than half of the reports on BRONJ with I.V. BP treatment were recorded within 3 years (Figs. 2 and 3). Our results indicate the importance of comparing drug safety profiles using post-marketing real-world data.

Gender was reported as a demographic and systematic risk factor for BRONJ [17]. BRONJ is rare in male patients with osteoporosis [18]. For oral BPs, the number of reports of BRONJ in males was lower than that in females (Table 1). Since zoledronate was used to prevent skeletal fractures in patients with multiple myeloma and prostate cancer, the number of reports for zoledronate in males might be similar to that in females (Table 1). Cumulative BP exposure has been proposed as an important risk factor for the development of BRONJ. There are various BP formulations and dosages. BPs were initially administered orally either once daily (alendronate [5 mg], risedronate [2.5 mg]), once weekly (alendronate [35 mg] and risedronate [17.5 mg]), and, recently, intravenous formulations (zoledronate [4 mg], pamidoronate [30–45 mg], and pamidoronate [90 mg]) has been developed. The median values for BRONJ caused by alendronate (35 mg, 680 days) were almost half that for alendronate (5 mg, 1263 days) (Table 4). We believe that the findings of this study will be of great value to clinicians who administer BPs to patients.

Several observational studies have identified potential risk factors associated with the development of BRONJ. Age, gender, cancer diagnosis with or without osteoporosis, renal dialysis, obesity, and co-administrated drug were reported as demographic and systematic risk factors for BRONJ [17]. Dentoalveolar surgeries such as extraction and dental implant placement are local risk factors [2, 3]. Corticosteroid use is associated with an increased risk for BRONJ [3]. These covariates should be confirmed by using further epidemiological studies, such as case–control or cohort studies.

SRS, such as the JADER database, is subject to various biases, including over-reporting, under-reporting, missing data, the exclusion of healthy individuals, the lack of denominator, and confounding factors [5, 10, 19, 20]. In the JADER database, some of the numbers of reports may reflect duplicate reporting due to factors such as follow-up reports received on a case or different persons reporting on the same patient case. It is recommended that identify duplicate reports of the same patient that come from different reporting sources and excluded these from the analysis. However, there is no key code for the identification of duplicate reports in the JADER database. Further studies are necessary to determine more precisely. Because of these limitations, disproportionality measures (ROR) do not allow for risk quantification. Rather, RORs offer a rough indication of signal strength and are only relevant to the *hypothesis*. In absolute terms, the ROR indicates an increased risk of adverse event reporting, and not a risk of adverse event occurrence. Therefore, careful attention must be paid to the interpretation of the results from the JADER database.

Despite the limitations inherent to SRS, our study indicated that BRONJ with BP treatment typically occurred within 3 years from the start of the treatment with BPs. Despite the clinical correlation between BPs and BRONJ, a definitive causal relationship has yet to be established [3]. There is no way to predict which individuals taking BPs are at greatest risk of developing BRONJ, nor is there evidence of prognostic indicators that are predictive of outcomes. Therefore, clinicians should comply with guidelines and monitor patients for adverse BRONJ events. This study was the first to evaluate the relationship between BP and BRONJ by using the JADER database. We hope that these data will update the information available to clinicians and be potentially useful for improving the management of BRONJ.

### Conclusion

This study was the first to evaluate the relationship between BPs and BRONJ by using the JADER database. The present analysis demonstrate that the incidence of BRONJ with BP treatment should be closely monitored for a 3-year period. WSP was deemed to be a useful tool for the time-to-onset analysis. We hope that these data will update the information available to clinicians and be potentially useful for improving the management of BRONJ induced by BPs.

## Abbreviations

IV: Intraveneous
BPs: Bisphosphonates
BRONJ: Bisphosphonate-Related Osteonecrosis of Jaw
AAOMS: American Association of Oral and Maxillofacial Surgeons
PMDA: the Pharmaceuticals and Medical Devices Agency
SRS: the Spontaneous Reporting System
JADER: the Japanese Adverse Drug Event Report
ADR: Adverse drug reaction
MedDRA: the Medical Dictionary for Regulatory Activities
ROR: Reporting Odds Ratio
CI: Confidence Intervals
WSP: Weibull Shape Parameter
